# Autonomy Support in Toddlerhood: Similarities and Contrasts Between Mothers and Fathers

**DOI:** 10.1037/fam0000450

**Published:** 2018-10

**Authors:** Claire Hughes, Anja Lindberg, Rory T. Devine

**Affiliations:** 1 Centre for Family Research, University of Cambridge; 2 School of Psychology, University of Birmingham

**Keywords:** parental autonomy support, infants, measurement invariance, parent–child gender

## Abstract

Infant exploration often hinges on parental autonomy support (i.e., parental behaviors that support children’s goals, interests, and choices), a construct that is widely applied in family studies of school-age children and adolescents but less studied in infants and toddlers. Notable gaps concern the equivalence, similarities, and contrasts between mothers’ and fathers’ autonomy support and the correlates of individual differences in autonomy support. To address these underresearched topics, we conducted parallel home-based structured play observations of 195 infants (*M*_age_ = 14.42 months, *SD* = .59) in dyadic interaction with mothers and fathers. Confirmatory factor analyses demonstrated measurement invariance across parent gender, enabling comparisons that revealed significantly moderately higher levels of autonomy support in mothers than in fathers. Individual differences in autonomy support were unrelated to either parental personality or child temperament, highlighting the potential importance of dyadic characteristics. Consistent with this view, whereas maternal autonomy support did not differ by child gender, fathers with sons displayed less autonomy support than did fathers with daughters.

Infants are wonderful explorers but not in a vacuum; rather, they explore with encouragement, support, and guidance from caregivers. According to self-determination theory, autonomy, competence, and relatedness underpin psychological health and effective engagement with the world (Deci & Ryan, 2015). Within this framework, caregiver behaviors that support children’s goals, interests, and choices are referred to as autonomy support (Whipple, Bernier, & Mageau, 2011). Suggesting a close overlap with the concept of parental scaffolding of preschool and school-age children’s problem-solving (for a recent review, see Mermelshtine, 2017), autonomy support in infancy and the preschool years each predict later executive function (e.g., Bernier, Carlson, & Whipple, 2010; Hughes & Devine, 2017b). It is interesting, however, that although the desire for autonomy increases markedly in the second year of life (Yarrow, Morgan, Jennings, Harmon, & Gaiter, 1982), autonomy support in this intervening period of toddlerhood has been little studied. More striking, however, is the lack of attention to fathers’ autonomy support. To address these twin gaps, the current study investigated (a) similarities and contrasts in the nature and/or mean level of mothers’ and fathers’ autonomy support in toddlerhood and (b) the correlates of individual differences in parents’ autonomy support.

## Autonomy Support and Scaffolding of Children’s Goal-Directed Activity

Developmental science has transformed the understanding of young children’s social and cognitive competencies, leading to a shift in the focus of research on early parental influences. For example, whereas parenting studies have long focused on general constructs such as sensitivity or authoritative discipline, interest in young children’s executive functions (Hughes & Devine, 2017a) has led to multidimensional models and a renewed interest in specific constructs, such as autonomy support. Studies involving samples of different ages and from different countries (Bernier, Carlson, Deschênes, & Matte-Gagné, 2012; Bernier et al., 2010; Hughes & Devine, 2017b; Hughes & Ensor, 2009) have shown that both global measures of autonomy support and more specific measures of parental scaffolding predict gains in preschoolers’ executive functions, even when background parental measures are controlled. Recent evidence that early parental autonomy support predicts children’s later academic success (Bernier, McMahon, & Perrier, 2017; Devine, Bignardi, & Hughes, 2016) has also highlighted the importance of early variation in parental autonomy support.

## Do Mothers and Fathers Provide Similar Kinds and Levels of Autonomy Support?

Despite fathers’ having become increasingly involved in child care (e.g., Sullivan, Coltrane, McAnnally, & Altintas, 2009), they have, with recent exceptions (Mills-Koonce et al., 2015; Nordahl, Zambrana, & Forgatch, 2016; Sethna et al., 2017), generally been overlooked in studies of individual differences in early parent–child interactions. Whereas early theorists (e.g., Belsky, Gilstrap, & Rovine, 1984) argued that differences in parental experience and/or context were likely to yield qualitative differences between mothers’ and fathers’ caregiving, recent theoretical reviews have called for gender-neutral models of parenting (e.g., Fagan, Day, Lamb, & Cabrera, 2014). Establishing the conceptual equivalence of maternal and paternal measures requires confirmatory factor analyses (CFAs) to test whether the model retains a good fit even when specific aspects (e.g., structure, variance, intercepts) are constrained to be equal across parent gender.

In turn, CFAs require larger samples than do those traditionally used in observational research. Indeed, just one previous observational study has tested measurement invariance in mothers’ and fathers’ caregiving. Applying CFAs of observational ratings of parental detachment, stimulation, positive regard, and animation at 6 and 24 months for 630 low-income North American families, Mills-Koonce et al. (2015) reported statistically equivalent overall ratings for mothers and fathers. That said, combining different aspects of parenting, different contexts, and different time points may have masked subtle nonequivalencies. Our first goal was thus to assess across-parent invariance for one specific measure (autonomy support) in one context and time point (an inset puzzle at 14 months).

## Parental Autonomy Support: Individual and Dyadic Correlates

Framed by Belsky’s (1984) seminal account of the multifaceted determinants of parenting, a further goal was to examine autonomy support in relation to both parental personality and child gender and temperament. Meta-analytic results have indicated small but consistent links between personality and parenting (Prinzie, Stams, Deković, Reijntjes, & Belsky, 2009) that appear similar in strength and nature for mothers and fathers (de Haan, Prinzie, & Deković, 2009). However, widespread reliance on single-informant self-report methodologies limits these conclusions. Moreover, the few studies that included direct observations of parent–infant interactions (e.g., Clark, Kochanska, & Ready, 2000; Karreman, van Tuijl, van Aken, & Deković, 2008) focused on parental control and responsiveness or sensitivity rather than on autonomy support. To our knowledge, only one study involving mothers of preschoolers (Neitzel & Stright, 2004) has reported a link between parental personality and observational ratings of autonomy support. Thus, our study adds to the field by involving both mothers and fathers of infants.

Turning to child characteristics, evidence has suggested a dosage effect in which children’s difficult behavior exerts a stronger influence on mothers’ than on fathers’ parenting (e.g., Elam, Chassin, Eisenberg, & Spinrad, 2017; Meunier, Roskam, & Browne, 2011). The gender composition of the parent–infant dyad has also appeared to be important. For example, in a study of parents and toddlers, Lovas (2005) reported a steady decline in parents’ emotional availability across mother–daughter, mother–son, father–daughter and father–son dyads. Likewise, whereas a meta-analysis of 126 observational studies of parents’ autonomy-support behaviors showed negligible effects of child gender (Endendijk, Groeneveld, Bakermans-Kranenburg, & Mesman, 2016), moderation analyses revealed significant effects of both time and age. Specifically, studies from the 1970s and 1980s reported greater autonomy support for sons than daughters, but studies from the 1990s onward reported greater autonomy support for daughters than for sons (B = 0.01, 95% CI [0.00, 0.01], *p* < .01). In addition, across all studies, parents showed more autonomy support for girls than for boys at the youngest age level (0–2 years). Not yet known, however, is whether mothers and fathers of infants are equally likely to provide greater autonomy support for daughters than for sons.

In sum, the current study applied parallel home observations of mothers’ and fathers’ autonomy support for 14-month-old infants in structured dyadic play to (a) assess their similarity in form and mean levels and (b) examine individual differences in relation to parental and child factors (e.g., age, personality temperament, gender).

## Method

### Participants

Participants were recruited to the New Fathers and Mothers Study, which aimed to examine links between parental well-being, parent–child interactions, and child outcomes in families with firstborn children. Eligibility hinged on the following criteria: first-time parents with no history of severe mental illness (e.g., psychosis) or substance misuse, expecting a healthy singleton baby, and planning to speak English as the primary language. We recruited 213 expectant mothers (primarily from ultrasound scans at a regional maternity hospital in the East of England but also from local “nearly new” sales and antenatal classes). Of the 205 families eligible for follow-up when the infants were 4 months old, 196 (96%) agreed to a home visit. At 14 months, two families declined to take part, but one family that missed the 4-month visit participated. Thus, 195 families took part when their infants (108 boys, 87 girls) were 14 months old (*M*_age_ = 14.42 months, *SD* = .59, range = 13.10–18.40; 98.5% were between 13.10 and 15.83 months, and three outliers were retained to maximize sample size). At the birth of their child, mothers were on average 32.61 years of age (*SD* = 3.60, range = 25.10–43.15) and fathers were on average 33.98 (*SD* = 4.35, range = 24.05–49.63). Both mothers and fathers had high levels of educational attainment: 84.6% of mothers and 77.1% of fathers had an undergraduate degree or higher. Mothers (60.8%) and fathers (61.4%) were drawn predominantly from professional occupations.

### Procedure

The National Health Service (United Kingdom) Research Ethics Committee study approved the protocol. In the last trimester, expectant parents completed an online questionnaire and in-person interview. Parents completed online questionnaire and 1-hr home visits at 4 months (not described here) and 14 months. At 14 months each parent was observed interacting with the infant in structured play followed by a free-play task (with mother–father order counterbalanced across visits and infants given rest breaks if needed).

### Measures

#### Parental demographics

Parents completed the Ladder of Subjective Social Status (Singh-Manoux, Adler, & Marmot, 2003), in which their placement on a 10-rung ladder was used to indicate their self-perceived education, income, and employment. Parental occupations were classified into one of nine standard groups (Office for National Statistics, 2010). At 14 months, parents reported on how many half days per week they had sole responsibility for their child, with responses scored on a 0–14 scale.

#### Parental autonomy support

Mothers and fathers were filmed with their child in separate 4-min dyadic sessions involving one of two Melissa & Doug eight-piece inset jigsaw puzzles (“Farm Friends”/“Vehicles” Large Peg Puzzle; see the online supplemental materials), counterbalanced across parents. Researchers asked parents to work with their children to complete the task and to bring them back to the activity if they went off task. To minimize distraction, the researcher left the room during the interaction. The Autonomy Support Coding manual (Whipple et al., 2011) was used to code each video. Parents were rated on a scale from 1 (*not autonomy supportive*) to 5 (*very autonomy supportive*). This indicated the degree to which they (a) provided appropriately tailored help (Concern for Competence); (b) used hints, instructions, and encouragement (Verbalisations); (c) kept their child on-task (Flexibility and Perspective Taking); and (d) involved the child as an active participant (Following Child’s Pace and Providing Opportunities for Choice). Supporting the validity of this rating procedure, Autonomy Support ratings have shown positive correlations with indicators of parenting quality, such as parental mind–mindedness, attachment representations, and sensitivity (e.g., Bernier et al., 2010; Bernier, Matte-Gagné, Bélanger, & Whipple, 2014) but have shown unique predictive associations with children’s later cognitive and social development (e.g., Bernier et al., 2012; Matte-Gagné, Bernier, & Lalonde, 2015). An experienced doctoral researcher trained three graduate researchers on a subsample of 20 videos. Note that the Autonomy Support coding scheme does not have an international reliability set, and the authors do not provide training. Instead, the coding team was trained against the lead coder’s ratings. The 20 training cases were selected by the lead coder to capture a wide range of performance. After detailed discussion and feedback on these cases, the four coders independently coded a reliability set of 15 mothers and 15 fathers. Interrater reliability was acceptable for each domain of autonomy support ratings (.70 < intraclass correlation < .81). Throughout the coding process, cases were regularly selected for discussion with the lead coder to prevent coder drift. Coders never coded mothers and fathers from the same family and were unfamiliar with the parents and children in each video.

#### Parental personality

Mothers and fathers completed the Ten Item Personality Inventory (TIPI; Gosling, Rentfrow, & Swann, 2003). Although the TIPI has typically exhibited poor internal consistency because there are only two 1- to 7-point items in each dimension, test–retest reliability over a 6-week period has been excellent (Gosling et al., 2003). Moreover, supporting its validity, the TIPI subscales have shown strong correlations (*r*s > .65) with subscales on lengthier personality questionnaires (Gosling et al., 2003). Averaging across the two items for each dimension yielded five 1- to 7-point scores representing Extraversion, Agreeableness, Conscientiousness, Emotional Stability, and Openness to Experience.

#### Child characteristics

Mothers completed the 36-item Early Childhood Behavior Questionnaire (ECBQ; Putnam, Gartstein, & Rothbart, 2006) to provide 1- to 7-point ratings on three dimensions of temperament: Negative Affect, Surgency, and Effortful Control. Ratings from the 12 pertaining to Negative Affect (e.g., “How often did your child become sadly tearful?”), Surgency (e.g., “How often did your child seem full of energy, even in the evening?”), and Effortful Control (e.g., “How often did your child wait patiently?”) were averaged, excluding items rated as *not applicable.* Negative Affect scores showed low internal consistency (α = .26) and so were dropped from analyses. Scores for Surgency and Effortful Control showed moderate internal consistency (α = .66 and .63, respectively).

### Analytic Strategy

Using a latent variable modeling framework in Mplus (Muthén & Muthén, 2017), we first examined the latent factor structure of ratings for mothers’ and fathers’ autonomy support. Next, using nested model comparisons, we examined the measurement invariance of each latent factor across mothers and fathers before testing latent mean differences between mothers and fathers (Brown, 2015). We then examined covariates of maternal and paternal autonomy support using multiple indicators, multiple causes models (Brown, 2015; Kline, 2012). Because the ratings had nonnormal distributions, we used a robust maximum likelihood estimator for each of our models (Kline, 2012) and three standard criteria: comparative fit index (CFI) of >.90, Tucker–Lewis index (TLI) of >.90, and a root-mean-square error of approximation (RMSEA) of <.08 (Brown, 2015) to evaluate model fit.

Almost all mothers (*N* = 194) and most fathers (*N* = 188) had complete and valid observational data. Missing questionnaire data did not exceed 5% for mothers or 10% for fathers. The electronic format prevented nonresponse to individual items. To avoid loss of data, we used a full information approach so that all families who participated in the 14-month home visit (*N* = 195) could be included in the analysis. Model parameters and standard errors were estimated in Mplus using all available data (Enders, 2011).

## Results

### Descriptive Statistics

As illustrated in Table 1, paired *t* tests showed differences between mothers and fathers for age, number of half days solely responsible for their child, emotional stability, and agreeableness. In contrast, mothers and fathers did not differ in the proportions: (a) with school–vocational qualifications (mothers: 15.4%; fathers: 22.9%), undergraduate degree (mothers: 41.5%; fathers: 38%), or postgraduate qualifications (mothers: 43.1%; fathers: 39.1%), χ^2^(2, *N* = 387 [195 mothers, 192 fathers]) = 3.551, *p* = .169, and (b) employed in service–elementary occupations (mothers: 6.2%; fathers: 3.7%), skilled trades–administration (mothers: 33%; fathers: 34.9%), or professional–managerial occupations (mothers: 60.8%; fathers: 61.4%), χ^2^(2, *N* = 383 [194 mothers, 189 fathers] χ^2^ = 1.30, *p* = .522). Boys and girls did not differ in age, effortful control, or surgency and were equally likely to go off task with mother (78.7% of boys vs. 76.7% of girls), χ^2^(1, *N* = 194) = .11, *p* = .744, and father (78.5% of boys vs. 80.7% of girls), χ^2^(1, *N* = 190) = .14, *p* = .707.[Table-anchor tbl1][Table-anchor tbl2]

### Measuring Parental Autonomy Support in Mothers and Fathers

Table 3 shows the correlation matrix for each of the autonomy support indicators for fathers and mothers (above and below the diagonal, respectively). First, we specified a model in which in each autonomy support indicator loaded onto a single latent factor. This provided an acceptable fit to the data in both mothers, χ^2^(2, *N* = 194) = 1.78, *p* = .41, RMSEA = 0, 90% confidence interval (CI) [0, .14], CFI = 1.00, TLI = 1.00, and fathers, χ^2^(2, *N* = 188) = 3.73, *p* = .15, RMSEA = .07, 90% CI [.00, .17], CFI = .994, TLI = .983. Factor score determinacy coefficients ranged from 0 to 1, with high scores (>.80) indicating good internal consistency (Brown, 2015). The coefficients for the autonomy support latent factor were .941 in mothers and .940 in fathers.[Table-anchor tbl3]

Next, we examined the measurement invariance of the autonomy support latent factor across mothers and fathers. Given the nonindependence of data from mothers and fathers, we used a repeated-measures modeling approach (typically applied in longitudinal measurement invariance testing) instead of a multiple-groups confirmatory factor analysis (Brown, 2015). Model parameters were considered noninvariant if there was a significant decrease in model fit indicated by the Satorra-Bentler scaled chi-square difference test (which is suitable for comparing nested models estimated using robust maximum likelihood) and decreases in CFI >.002 (Meade, Johnson, & Braddy, 2008). We also calculated an effect size (*w*) to estimate the magnitude of differences in model fit, where *w* = .1, .3, and .5 represented small, medium, and large effects, respectively (Newsom, 2015). When model constraints produced changes in both chi-square and CFI, we released these constraints. We followed procedures for evaluating longitudinal measurement invariance specified by Brown (2015), Geiser (2013), and Newsom (2015).

**Figure 1 fig1:**
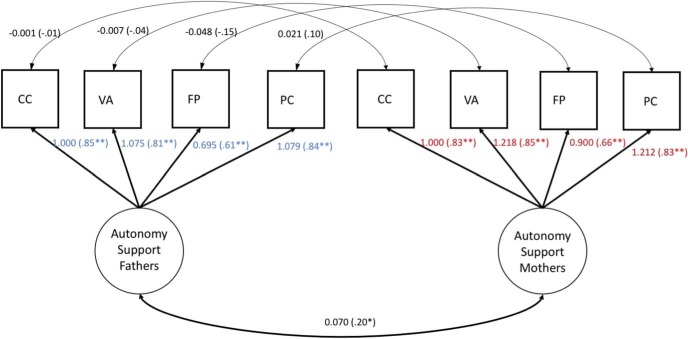
Unstandardized and standardized (in parentheses) estimates for the equal form autonomy support model. CC = Concern for Competence; VA = Verbalisations; FP = Flexibility and Perspective Taking; PC = Following Child’s Pace and Providing Opportunities for Choice. * *p* < .05. ** *p* < .01.

Table 4 presents the model fit indices for each of these models. First, we tested the assumption of equal form by specifying a two-factor model in which each autonomy support indicator loaded onto two latent factors (i.e., one for mothers and one for fathers). To identify the model, we set the lead indicators of the maternal and paternal autonomy support latent factors to 1. To estimate latent factor means for maternal and paternal autonomy support, which Mplus sets at 0 as a default, we set the lead indicator intercept to 0 to identify the latent factor means (Brown, 2015; Geiser, 2013). We permitted each maternal indicator of autonomy support to covary with the corresponding paternal indicator to account for any indicator-specific correlations across mothers and fathers (Brown, 2015; Model 1). Figure 1 presents standardized and unstandardized estimates for this model, which showed a good fit to the data, indicating configural invariance (i.e., the same latent factor structure existed in mothers and fathers; Newsom, 2015).[Table-anchor tbl4][Fig-anchor fig1]

Next, we tested the assumption of equal factor loadings by constraining the corresponding factor loadings for the autonomy support latent factors to equality across mothers and fathers (Model 2). These constraints did not reduce model fit, demonstrating that associations between the autonomy support indicators and the latent factor were similar in mothers and fathers (i.e., the models showed metric invariance; Newsom, 2015). Then we tested the assumption of equal indicator intercepts by constraining the indicator intercepts to equality across mothers and fathers. This constraint reduced the overall model fit (Model 3). To determine which indicator resulted in the reduction of fit, we constrained one indicator at a time, starting with Verbalisations (Models 4 and 5). Note that the intercepts for Concern for Competence were set to 0 in both mothers and fathers to identify the model making these intercepts invariant (Geiser, 2013). Because the intercept constraints on Verbalisations and Flexibility and Perspective Taking did not produce decreases in model fit, this meant that the intercepts for Following Child’s Pace and Providing Opportunities for Choice were noninvariant. We therefore released this constraint for future models and used Model 5 as the baseline for comparison for Model 6. Intercepts for Following Child’s Pace and Providing Opportunity for Choice were lower for mothers (*b* = −.483) than for fathers (*b* = −.339). That is, compared with fathers, mothers were more likely to be rated as respecting their child’s pace and allowing their child an active role in the interaction for reasons other than autonomy support.

Next, we tested the assumption of equal indicator error variances by constraining the error terms to equality across mothers and fathers one indicator at a time (Models 6, 7, 8, and 9). Constraining the error variances for Flexibility and Perspective Taking (Model 8) and for Following Child’s Pace and Providing Opportunities for Choice (Model 9) reduced overall model fit (relative to Model 7), and so we released the constraints on these parameters. To avoid eliminating noninvariant items, we retained these items in the model but freely estimated the noninvariant parameters across mothers and fathers. This method is preferable because it uses all available data and reduces bias (Byrne & Campbell, 1999). Together our models demonstrated that the autonomy support latent factor exhibited partial measurement invariance when applied to mothers and fathers in the same family (Geiser, 2013). That is, although there was support for configural and metric invariance, one indicator intercept and two indicator error variances were not invariant. That said, the remaining parameters of the model were invariant across mothers and fathers, suggesting that the measure of autonomy support functioned equivalently when applied to mothers and fathers.

### Comparing Mothers’ and Fathers’ Autonomy Support

Having established partial measurement invariance, we next examined latent mean differences between mothers and fathers in autonomy support. To this end, we set the latent factor variances for maternal and paternal autonomy support to be equal (Model 10) and compared the fit of this constrained model against the partial measurement invariance model (Model 7). The corrected Δχ^2^ suggested a decrease in model fit with a small effect size. However, because the CFI did not decrease by more than .002, we maintained this constraint on the model. Next, we constrained the autonomy support latent factor means to be equal across mothers and fathers and compared the fit of this model (Model 11) to Model 10. The resultant small- to medium-sized decrease in model fit indicated that the autonomy support latent factor means were significantly different. The results from Model 10 indicated that mothers (*b* = 3.02, *SE* = .05) exhibited significantly higher levels of autonomy support than did fathers (*b* = 2.82, *SE* = .05), corrected Δχ^2^(1) = 9.32, *p* = .002, *w* = .22.

### Correlates of Mothers’ and Fathers’ Autonomy Support

**Figure 2 fig2:**
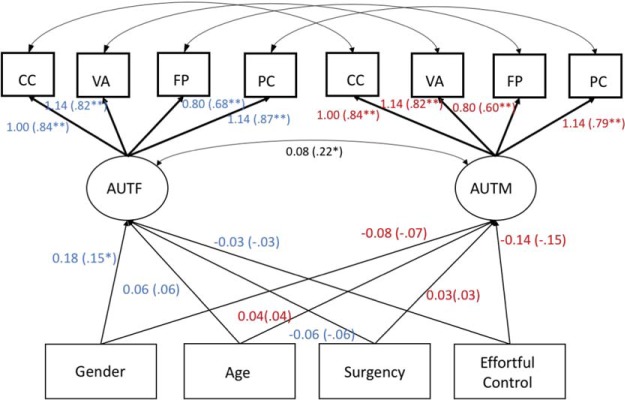
Unstandardized and standardized (in parentheses) estimates for child correlates of maternal and paternal autonomy support. To simplify the presentation, the path model diagram omits parameter estimates for covariances between indicator residuals and between gender, age, surgency, and effortful control. Lighter data indicate correlates of paternal autonomy support, and darker data indicate correlates of maternal autonomy support. CC = Concern for Competence; VA = Verbalisations; FP = Flexibility and Perspective Taking; PC = Following Child’s Pace and Providing Opportunities for Choice; AUTF = autonomy support in fathers; AUTM = autonomy support in mothers. * *p* < .05. ** *p* < .01.

Next, we used structural equation modeling to examine the correlates of mothers’ and fathers’ autonomy support. Our first set of models regressed equality-constrained latent variables for parental autonomy support (Model 10) onto child gender (1 = boys, 2 = girls), child age, surgency, and effortful control (see Figure 2). The model fit was acceptable, χ^2^(50, *N* = 195) = 62.78, RMSEA = .036, 90% CI [0, .062], CFI = .979, TLI = .974. Neither surgency nor effortful control was significantly associated with either maternal or paternal autonomy support. Gender was significantly associated with autonomy support in fathers (only), with paternal autonomy support being greater for daughters than for sons. When we constrained the corresponding gender paths to be equal across mothers and fathers, the overall model fit decreased significantly, χ^2^(51, *N* = 195) = 67.27, RMSEA = .04, 90% CI [.01, .065], CFI = .973, TLI = .968, corrected Δχ^2^(1, *N* = 195) = 4.49, *p* = .034, ΔCFI = .006, *w* = .15, indicating that the effect of child gender was marginally greater for fathers than for mothers.[Fig-anchor fig2]

**Figure 3 fig3:**
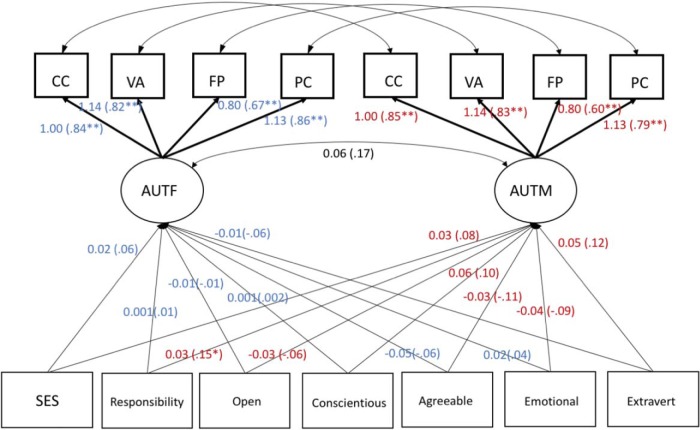
Unstandardized and standardized (in parentheses) estimates for parental correlates of maternal and paternal autonomy support. To simplify the presentation, the path model diagram omits parameter estimates for covariances between indicator residuals and between socioeconomic status, responsibility, and personality indicators. Lighter data indicate correlates of paternal autonomy support, and darker data indicate correlates of maternal autonomy support. CC = Concern for Competence; VA = Verbalisations; FP = Flexibility and Perspective Taking; PC = Following Child’s Pace and Providing Opportunities for Choice; AUTF = autonomy support in fathers; AUTM = autonomy support in mothers; SES = socioeconomic status. * *p* < .05. ** *p* < .01.

In our second set of models, we examined correlates of autonomy support in mothers and fathers. Figure 3 shows the associations between dimensions of personality, socioeconomic status (SES), the number of half days per week in which each parent was solely responsible for caring for the child (responsibility), and autonomy support. The model fit was acceptable, χ^2^(121, *N* = 197) = 152.25, RMSEA = .036, 90% CI [.013, .053], CFI = .954, TLI = .947. Inspection of the model parameters revealed that neither personality nor parental SES was correlated with parental autonomy support in mothers or fathers. There was a weak positive association between mothers’ (but not fathers’) responsibility and autonomy support. When we constrained the responsibility–autonomy support path to be equal for mothers and fathers, the overall model fit did not degrade, χ^2^(122, *N* = 197) = 153.31, RMSEA = .036, 90% CI [.012, .053], CFI = .954, TLI = .947, corrected Δχ^2^(1, *N* = 197) = 1.06, *p* = .303, ΔCFI = 0, *w* = .07, indicating that these paths did not differ significantly in strength for mothers and fathers.[Fig-anchor fig3]

## Discussion

This study of 195 infants 14 months old filmed at home in parallel structured dyadic play sessions with mothers and fathers contributes to the field in two ways. First, testing observational ratings of maternal and paternal autonomy support for measurement invariance was a relatively novel feature of the study, which supported the validity of comparing latent factor scores for mothers’ and fathers’ autonomy support: On average, mothers displayed higher levels of autonomy support than did fathers. Second, autonomy support was unrelated to parental personality or child temperament but did differ according to the gender composition of the parent–toddler dyad. Specifically, whereas mothers of daughters and mothers of sons showed similar levels of autonomy support, fathers of sons showed less autonomy support than did fathers of daughters. Next, we discuss these two sets of findings in turn.

### Autonomy Support Is Equivalent in Mothers and Fathers but Higher in Mothers

As noted in the introduction, tests of measurement invariance are of vital importance for establishing conceptual clarity within family research (Dyer, 2015) but require relatively large samples and so have typically been restricted to survey-based studies. As noted in the introduction, whereas early studies of father–child interactions emphasized differences between mothers and fathers, later empirical evidence showed that similarities typically eclipse contrasts, such that gender-neutral models of parenting are needed. However, studies that tested measurement invariance have suggested a more nuanced picture. For example, in an early study to adopt this approach, Adamsons and Buehler (2007) surveyed 416 cohabiting heterosexual couples with a child in sixth grade and showed that although the two negative scales (harshness and intrusiveness) were equivalent for mothers and fathers, the positive scale (acceptance) was not. In other words, mothers and fathers appeared alike in their negative parenting behaviors but differed in the nature of their supportive behaviors.

In contrast, the four indicators of autonomy support in the current study showed similar patterns of association (in terms of the structure and strength of relationship) with the latent factor in mothers and fathers. This partial measurement invariance (i.e., configural and metric invariance but not intercept or error invariance) indicates conceptual equivalence in ratings of autonomy support for each parent. Thus, this scale can be applied fairly to both mothers and fathers, enhancing the validity of comparisons of latent factor means. It is interesting that findings from the only other study to test configural measurement invariance (i.e., equal form, factor loadings, and factor variances) in observational measures of mothers’ and fathers’ interactions with young children also support the overall equivalence of latent measures (Mills-Koonce et al., 2015). Several differences between the measures used to construct latent variables in the current study and this prior observational study deserve note. Specifically, whereas latent scores for autonomy support in the current study were based on parallel home observations of mothers and fathers in dyadic structured play with their 14-month-old infants, Mills-Koonce et al. (2015) compared composite indices that encompassed different time points and multiple facets of parenting. It is therefore striking that studies adopting either fine-grained or broad-brushstroke approaches provide convergent evidence for partial measurement invariance between mothers and fathers. Note, however, that in each study at least one indicator was not invariant between mothers and fathers; this suggests that creating composite scores (rather than latent variables) might give rise to misleading results when comparing mothers and fathers (Newsom, 2015).

The similarity of results from these distinct approaches raises an interesting question regarding relations between autonomy support and other aspects of parenting. Indeed, some theorists (e.g., Tamis-LeMonda, Kuchirko, & Song, 2014) have proposed that scaffolding (a construct that is closely related to autonomy support) is simply one example of maternal responsiveness. This view is supported by reports of stable individual differences in parental verbal responsiveness across structured and unstructured contexts, despite elevated talk to infants in structured play (Tamis-LeMonda, Kuchirko, Luo, Escobar, & Bornstein, 2017). Further evidence to support a unitary measure of “positive involvement” comes from an observational study of 726 Norwegian fathers that included four distinct aspects of positive parenting that were quite similar to those adopted in the current study: sensitivity, engagement, positive regard, and stimulation (Nordahl et al., 2016). Crucially, however, the ratings for each aspect were averaged across free play, clean-up and structured play settings. As noted by Tamis-Lemonda et al. (2017), method effects are powerful; so more work is needed to conduct analyses based on context-specific measures to assess contrasts between autonomy support and other aspects of positive parenting, such as warmth or acceptance.

Consistent with previous findings (e.g.,Kwon & Elicker, 2012), the mothers in our study displayed higher levels of autonomy support than did fathers. One obvious explanation for this contrast hinges on the substantial difference between mothers and fathers in time spent alone with the infant (4.53 half days for mothers vs. .97 half days for fathers). Consistent with this view, variation in this responsibility measure was significantly related to variation in autonomy support for mothers (but not fathers, for whom there was limited variability, because nearly all fathers were in full-time employment). However, it is worth emphasizing that (a) the mean difference in autonomy support was quite modest (3.00/5 for mothers; 2.82/5 for fathers) and (b) constraining this path to equality between mothers and fathers did not reduce model fit. Thus, our findings are also consistent with an emphasis on quality rather than quantity of time with children. Alternatively, as suggested by interaction effects in a study involving triadic observations of young children with mothers and fathers (Volling, Blandon, & Gorvine, 2006), within-family processes (e.g., modeling, spillover) may also serve to narrow differences between mothers’ and fathers’ autonomy support.

### Do Child Factors Contribute to Variation in Parental Autonomy Support?

Our coding scheme focused on the fit between child and parent behavior and so should have taken variation in child behavior into account. However, children’s reactions to a situation may affect the ease with which parents can provide well-tuned responses. Findings from two lab-based studies have suggested that young children do indeed react differently to a specific situation when with their mother or father. In the first study, 26-month-old toddlers displayed less engagement and greater negativity toward fathers than mothers, especially in triadic interactions (Kwon & Elicker, 2012). In the second, preschoolers displayed more frequent attention-seeking behaviors toward fathers than mothers in a sibling jealousy paradigm (Volling et al., 2014). More work is needed to establish the factors that underpin such contrasts and to examine how they might contribute to differences in mean levels of maternal and paternal autonomy support.

Despite showing good variability, background measures of parental personality and infant temperament were unrelated to variation in autonomy support. That is, the goodness of fit between parent and child, rather than any absolute individual characteristic, may underpin variation in the quality of parent–child interactions (cf. Belsky, 1984). Even though adult ratings of temperament–off-task behavior did not differ by child gender, fathers of daughters showed greater autonomy support than did fathers of sons. Gendered parent–child activities have been reported in a large-scale study involving families from the United Kingdom, the United States, and Canada (Baker & Milligan, 2016), making it but a small step to suggest that child gender may also influence the *quality* of parent–child interactions.

Our findings add to this literature by indicating a greater impact of infant gender on fathers’ than mothers’ autonomy support. It is worth noting, however, that Nordahl et al.’s (2016) study of Norwegian fathers and their 1-year-old infants showed no effect of child gender on fathers’ positive involvement. Because this measure of positive involvement was the aggregate of average ratings of sensitivity, engagement, positive regard, and stimulation, it may be that gender stereotypes have more influence on fathers’ autonomy support than on other aspects of parenting. Alternatively, gender stereotyping may vary significantly between fathers from different countries. Of interest in relation to this point are recent findings from a study of 299 Dutch parents of 3-year-olds in which fathers with gender-stereotyped attitudes used more physical control with sons than with daughters, whereas fathers with counterstereotyped attitudes used more physical control with daughters than with sons (Endendijk et al., 2017). Between-studies differences in setting (home vs. lab) also deserve note. Given the emphasis on gender equality within Norwegian society, the public setting of a laboratory may have elicited more similar parental behaviors in fathers of infant sons and daughters. Testing these alternative accounts requires the same paradigms and settings to be applied in between-countries comparisons of parent–infant interactions.

### Study Limitations

Participants in the current study were relatively educated and affluent, raising questions about the generalizability of our results to more disadvantaged families. In a large-scale study of father–infant interactions, Nordahl et al. (2016) noted that fathers who took part in video observations were older and more educated than were fathers who declined; a similar selection bias in our sample is likely. However, because high SES parents typically report less traditional views than do low SES parents (Cabrera, Shannon, & Tamis-LeMonda, 2007), our findings may actually underestimate effects of child gender on fathers’ autonomy support.

### Future Directions

Our cross-sectional design precluded investigation of either the stability of individual differences in mothers’ and fathers’ autonomy support or the predictive utility of parents’ autonomy support in relation to children’s early executive functions. Recent longitudinal findings from a small-scale study of 46 low-risk families (Hertz, Bernier, Cimon-Paquet, & Regueiro, in press) have suggested that the quality of toddlers’ interactions with fathers shows potentially unique associations with kindergarten teachers’ ratings of executive function difficulties. Further work is needed to assess the independence and interplay between maternal and paternal autonomy support in toddlerhood as predictors of children’s later executive function performance. Addressing these questions will enhance the effectiveness of interventions to support young children to acquire the competencies they need to flourish.

## Supplementary Material

10.1037/fam0000450.supp

## Figures and Tables

**Table 1 tbl1:** Descriptive Statistics for Continuous Parent Measures

Variable	Mother			Father			*d*	*t* (*df*)^a^
	*M*	*SD*	Range	*M*	*SD*	Range		
Demographics and personality traits								
Age (years)	32.61	3.60	25.10–43.15	33.98	4.35	24.05–49.63	.40	5.45 (189)***
Responsibility (half days)	4.53	3.29	0–14	.97	1.88	0–10	.87	12.06 (190)***
Social standing	7.32	1.39	3–10	7.32	1.27	4–10	.02	.25 (173)
Openness to Experience	5.04	1.15	1–7	4.76	1.15	1.5–7	.16	1.89 (173)
Conscientiousness	5.55	1.11	2.5–7	5.22	1.23	1.5–7	.22	3.36 (173)**
Emotional Stability	4.51	1.41	1–7	5.15	1.26	1.5–7	.31	2.83 (173)**
Agreeableness	5.06	1.03	2–7	4.67	1.13	2–7	.26	4.12 (173)***
Extraversion	4.49	1.50	1–7	4.15	1.55	1–7	.14	2.09 (173)
Autonomy support								
Concern for Competence	3.02	.74	1–5	2.82	.67	2–5	.21	2.81 (187)**
Verbalisations	3.06	.87	1–5	2.84	.76	1–5	.18	2.50 (187)*
Flexibility and Perspective Taking	2.36	.81	1–5	2.14	.63	1–4	.24	2.75 (128)**
Following Child’s Pace and Providing Opportunities for Choice	2.99	.89	1–5	2.89	.74	1–5	.07	.93 (187)
^a^ *t* tests were paired.								
* *p* < .05. ** *p* < .01. *** *p* < .001.								

**Table 2 tbl2:** Descriptive Statistics for Continuous Children’s Measures

Measure	Girls			Boys			*d*	*t*(*df*)^a^
	*M*	*SD*	Range	*M*	*SD*	Range		
Age (months)	14.37	.70	13.10–18.40	14.46	.48	13.60–15.83	.15	1.01 (193)
ECBQ Surgency	4.35	.59	3.20–5.67	4.34	.61	2.25–5.50	.02	.13 (180)
ECBQ Effortful Control	4.21	.60	2.91–5.33	4.12	.64	2.38–5.45	.14	.97 (180)
*Note*. ECBQ = Early Childhood Behavior Questionnaire.								
^a^ *t* tests were independent.								

**Table 3 tbl3:** Correlations Between Parental Measures

Measure	1	2	3	4	5	6	7	8	9	10	11
1. Parent age	—	.16*	.21**	.15	−.04	.02	.02	−.12	−.06	.03	.002
2. Parent SES	.10	—	−.12	.09	−.04	−.11	−.04	.06	.05	.11	.04
3. Openness to Experience	.01	−.02	—	.22**	.08	.02	.26**	−.02	−.04	.01	−.04
4. Conscientiousness	.07	.01	.03	—	.20**	.14	.02	−.01	.08	.04	−.03
5. Emotional Stability	.07	−.03	.03	.25**	—	.31**	.03	−.02	−.01	.05	.04
6. Agreeableness	.09	−.04	.03	.08	.25**	—	.04	−.08	.07	−.18*	−.06
7. Extraversion	−.10	.001	.37**	.01	.13	−.04	—	−.08	.02	−.10	−.05
8. Concern for Competence	−.03	.11	.04	.04	−.10	−.14	.12	—	.70**	.49**	.71**
9. Verbalisations	−.05	.04	−.03	.10	−.08	−.11	.06	.71**	—	.37**	.68**
10. Flexibility and Perspective Taking	−.11	−.07	−.05	.10	−.05	−.13	.13	.54**	.50**	—	.48**
11. Following Child’s Pace and Providing Opportunities for Choice	.08	.11	−.02	−.03	−.08	−.14	.02	.66**	.72**	.49**	—
*Note*. Data for mothers appear below the diagonal, and for fathers, above the diagonal. SES = socioeconomic status.											
* *p* < .05. ** *p* < .01.											

**Table 4 tbl4:** Model Fit Indices

Model	χ^2^	*df*	scf	Δχ^2a^	Δ*df*	*w*^b^	RMSEA [90%CI]	CFI	ΔCFI	TLI
Measurement invariance										
Equal form (Model 1)	18.29	15	1.03				.034 [0, .079]	.994		.988
Equal loadings (Model 2)	19.75	18	1.09	1.93	3	.05	.022 [0, .069]	.997	.003	.995
Equal intercepts (Model 3; VA, FP, PC)	27.70	21	1.07	6.44*	1	.18	.041 [0, .078]	.987	−.011	.983
Equal intercepts (Model 4; VA)	19.99	19	1.08	.07	1	.02	.016 [0, .066]	.998	.001	.997
Equal intercepts (Model 5; VA, FP)	21.26	20	1.07	1.32	1	.08	.018 [0, .065]	.998	0	.997
Equal error variance (Model 6; CC)^c^	22.49	21	1.06	1.27	1	.08	.019 [0, .065]	.997	−.001	.996
Equal error variance (Model 7; CC, VA)	20.26	22	1.19	.07	1	.02	0 [0, .055]	1.000	.003	1.004
Equal error variance (Model 8; CC, VA, FP)	25.36	23	1.18	6.06*	1	.17	.023 [0, .065]	.995	−.005	.994
Equal error variance (Model 9; CC, VA, PC)^d^	26.84	23	1.16	14.05**	1	.27	.029 [0, .069]	.993	−.007	.991
Latent mean differences										
Equal latent factor variances (Model 10)^e^	23.75	23	1.18	4.08*	1	.14	.013 [0, .061]	.999	−.001	.998
Equal latent means (Model 11)	33.07	24	1.18	9.32**	1	.22	.004 [0, .078]	.983	−.016	.980
*Note*. scf = scaling correction factor; RMSEA = root-mean-square error of approximation; CI = confidence interval; CFI = comparative fit index; TLI = Tucker–Lewis index; CC = Concern for Competence; VA = Verbalisations; FP = Flexibility and Perspective Taking; PC = Following Child’s Pace and Providing Opportunities for Choice.										
^a^ Satorra-Bentler scaled chi-square difference test. ^b^ Contingency chi-square effect size. ^c^ Model 6 is compared against Model 5. ^d^ Model 9 is compared against Model 7. ^e^ Model 10 is compared against Model 7.										
* *p* < .05. ** *p* < .01.
