# Supplementary figures and images for: Autonomy Support in Toddlerhood: Similarities and Contrasts Between Mothers and Fathers
Source: J Fam Psychol. 2018 Oct;32(7):915–25. doi: 10.1037/fam0000450 (PMC6205245; doi:10.1037/fam0000450)

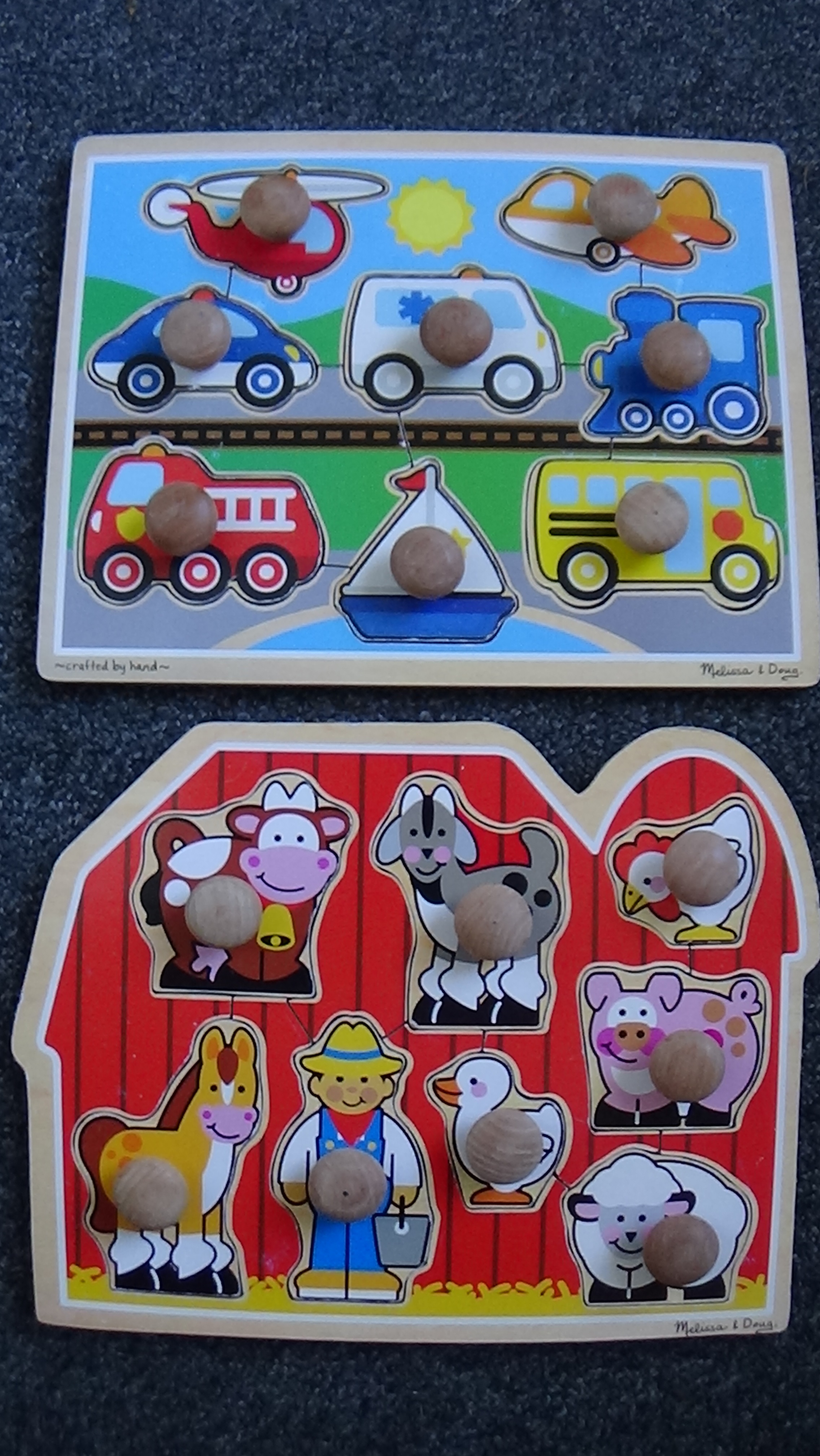

Supplement: Supplementary file 1 [file FAM-2017-1354Suppl.zip › InsetPuzzle_grey_background.jpg]
